# Self-disturbance in first-episode psychosis: Theoretical framework and potential cannabis interactions - a systematic review

**DOI:** 10.3389/fpsyt.2025.1733254

**Published:** 2026-01-05

**Authors:** Valerio Ricci, Domenico De Berardis, Giovanni Martinotti, Giuseppe Maina

**Affiliations:** 1 San Luigi Gonzaga Hospital, University of Turin, Orbassano, Italy; 2 Regione Gonzole, Orbassano, Teramo, Italy; 3 Department of Mental Health, Psychiatric Service for Diagnosis and Treatment, Hospital “G. Mazzini”, Teramo, Italy; 4 Department of Neurosciences, Imaging and Clinical Sciences, Università degli Studi G. D’Annunzio Chieti-Pescara, Chieti, Italy; 5 Department of Neurosciences “Rita Levi Montalcini”, University of Turin, Turin, Italy

**Keywords:** anomalous self-experience, cannabis, depersonalization, dissociation, ease, endocannabinoid system, first-episode psychosis, ipseity

## Abstract

Cannabis use represents a significant environmental risk factor for psychotic disorders, with emerging evidence suggesting complex interactions between cannabinoid exposure, dissociative experiences, and fundamental disturbances of self-experience (self-disorders) in early psychosis. This systematic review examines the phenomenological and neurobiological relationships among dissociation, cannabis use, and self-disturbance in first-episode psychosis (FEP) patients. Following PRISMA guidelines, we conducted a comprehensive search of four databases (PubMed, Scopus, PsycINFO, Web of Science) from January 1990 to September 2025, identifying 22 studies meeting inclusion criteria (total N = 3,847 participants). Results demonstrate that cannabis use, particularly high-potency THC products, is consistently associated with significantly elevated dissociative experiences compared to non-using FEP patients. Across studies employing the Dissociative Experiences Scale-II (DES-II), cannabis users showed score elevations of 11–13 points, exceeding clinically significant thresholds and persisting at follow-up assessments. Daily high-potency cannabis use was associated with three-fold increased odds of clinically significant dissociation (OR: 3.21, 95% CI: 2.14-4.82) and more severe anomalous self-experiences compared to non-using patients. Multiple mechanisms mediate these relationships: alterations in CB1 receptor availability and endocannabinoid system dysregulation in regions critical for self-awareness, disruption of neural networks supporting minimal self-consciousness, and acute psychotomimetic effects including depersonalization and derealization that may persist beyond intoxication periods. Cannabis-related dissociation shows distinct phenomenological characteristics compared to primary dissociative disorders, with greater self-world boundary confusion and more severe distortions of first-person perspective. Longitudinal studies indicate that persistent dissociative symptoms and self-disorders in cannabis-using FEP patients predict poorer functional outcomes (12-month GAF scores: 52 ± 14 *vs*. 67 ± 12 in non-users, p<0.001) and increased symptom chronicity. However, approximately 75% of patients showed dissociation reduction following cannabis cessation, suggesting potential reversibility. GRADE certainty of evidence was rated as moderate for dissociative symptom severity (downgraded for observational study designs) and low for self-disturbance outcomes (limited direct evidence with EASE assessment). This review highlights the importance of comprehensive phenomenological assessment in cannabis-using FEP patients, incorporating dissociative symptomatology and basic self-disturbance evaluation, with implications for early intervention strategies and targeted therapeutic approaches. Future research should employ longitudinal designs with repeated phenomenological assessments, biological verification of cannabis exposure, and integration of neuroimaging with experiential measures to elucidate causal mechanisms.

## 1 Introduction

The relationship between cannabis use and psychotic disorders represents one of the most extensively studied areas in psychiatric epidemiology, with converging evidence demonstrating dose-dependent associations between cannabinoid exposure and increased risk of psychosis onset (1–3). First-episode psychosis (FEP) constitutes a period of particular clinical significance, characterized by heightened vulnerability to both substance-related complications and long-term functional impairment. Cannabis use prevalence among FEP patients ranges between 30% and 70%, substantially exceeding general population rates (4–6).

Beyond the well-established association between cannabis and psychosis onset, emerging phenomenological research has identified specific experiential alterations that may characterize cannabis-associated psychosis. Dissociative experiences, encompassing depersonalization (altered sense of self) and derealization (altered perception of external reality), represent a dimension of psychopathology that has received relatively limited attention in cannabis-psychosis research, despite anecdotal clinical observations and experimental evidence suggesting significant cannabinoid-induced dissociative phenomena (7–9).

Parallelly, phenomenological psychiatry has reintroduced the concept of self-disorders (Selbststörungen) or disturbances of minimal self (ipseity) as potentially fundamental to schizophrenia spectrum pathology (10, 11). Self-disorders encompass anomalous self-experiences (ASEs) that reflect disturbances in the pre-reflective, tacit sense of existing as a unified, embodied subject of experience—what phenomenological philosophy terms the ‘minimal self’ or ipseity. These experiences, systematically assessed through instruments such as the Examination of Anomalous Self-Experience (EASE), aggregate specifically in schizophrenia spectrum disorders and have demonstrated predictive validity for psychosis onset (12, 13). Before examining the intersection of cannabis use with these phenomenological constructs, clarification of terminology is necessary given the multiple overlapping terms employed in this literature. “Self-disorders” (Selbststörungen in the original German phenomenological tradition) and “self-disturbance” are used interchangeably to describe anomalous alterations in basic self-experience characteristic of schizophrenia spectrum disorders (10, 11). At a more fundamental level, “minimal self” and “ipseity” refer to the pre-reflective sense of existing as a unified subject—the basic “I-ness” that precedes explicit self-reflection. Disturbances in minimal self/ipseity represent the theoretical mechanism proposed to underlie observable self-disorders (10). “Anomalous self-experiences” (ASEs) designate the specific experiential manifestations that are operationally assessed through instruments like EASE (11). Thus, the conceptual framework proceeds from ipseity/minimal self (fundamental selfhood) through ipseity disturbance (fundamental alteration) to self-disorders/self-disturbance (broad diagnostic category) and finally to anomalous self-experiences (specific measurable symptoms). While phenomenological literature often uses these terms interchangeably (13, 14), we maintain distinctions between theoretical constructs (ipseity), diagnostic categories (self-disorders), and operational assessments (ASEs/EASE) throughout this review.

The potential intersection of these three domains—cannabis use, dissociative experiences, and self-disturbance—remains inadequately characterized despite theoretical and clinical reasons to suspect meaningful interactions. Cannabis, particularly delta-9-tetrahydrocannabinol (THC), reliably produces acute dissociative phenomena including depersonalization and derealization in experimental settings (15: 16). Simultaneously, phenomenological descriptions of cannabis intoxication share features with descriptions of minimal self-disturbance, including alterations in temporal flow, embodiment, and sense of presence (17).

From a neurobiological perspective, the endocannabinoid system—particularly CB1 receptors—is densely distributed in brain regions implicated in both self-consciousness and reality monitoring, including the prefrontal cortex, insula, posterior cingulate cortex, and temporoparietal junction (18);. Recent neuroimaging studies have demonstrated altered CB1 receptor availability in FEP patients, with evidence of dysregulated endocannabinoid system function affecting networks critical for self-world distinction and phenomenological experience (19).

Despite these converging lines of evidence, systematic integration of cannabis effects, dissociative phenomena, and self-disturbance in early psychosis remains absent from the literature. Previous reviews have examined cannabis-psychosis relationships without phenomenological specificity (20), dissociation in psychosis without substance-use considerations (21), or self-disorders in schizophrenia without examining environmental precipitants (22). A recent systematic review examined relationships between cannabis and dissociation but concluded that methodological limitations and heterogeneity prevented definitive conclusions (23). Critically, this review did not specifically focus on FEP populations or incorporate the self-disturbance framework.

The clinical relevance of this integrated examination is amplified by several contemporary trends: increasing cannabis potency globally (24–26);, proliferation of high-THC synthetic cannabinoids, expanding legalization creating increased availability, and growing recognition that early intervention in psychosis requires attention to specific phenomenological vulnerability markers (27–29). If cannabis systematically influences dissociative experiences and self-disturbance in early psychosis, this has substantial implications for assessment, prognosis, and intervention strategies.

This systematic review aims to: (1) synthesize current evidence on relationships between cannabis use and dissociative experiences in FEP patients; (2) examine associations between cannabis use and self-disturbance/anomalous self-experience; (3) explore potential neurobiological and phenomenological mechanisms linking cannabinoid exposure to dissociation and self-disorders; (4) identify methodological strengths and limitations in existing literature; and (5) provide clinical implications for assessment and intervention in cannabis-using FEP patients.

## 2 Methods

**Figure 1:**
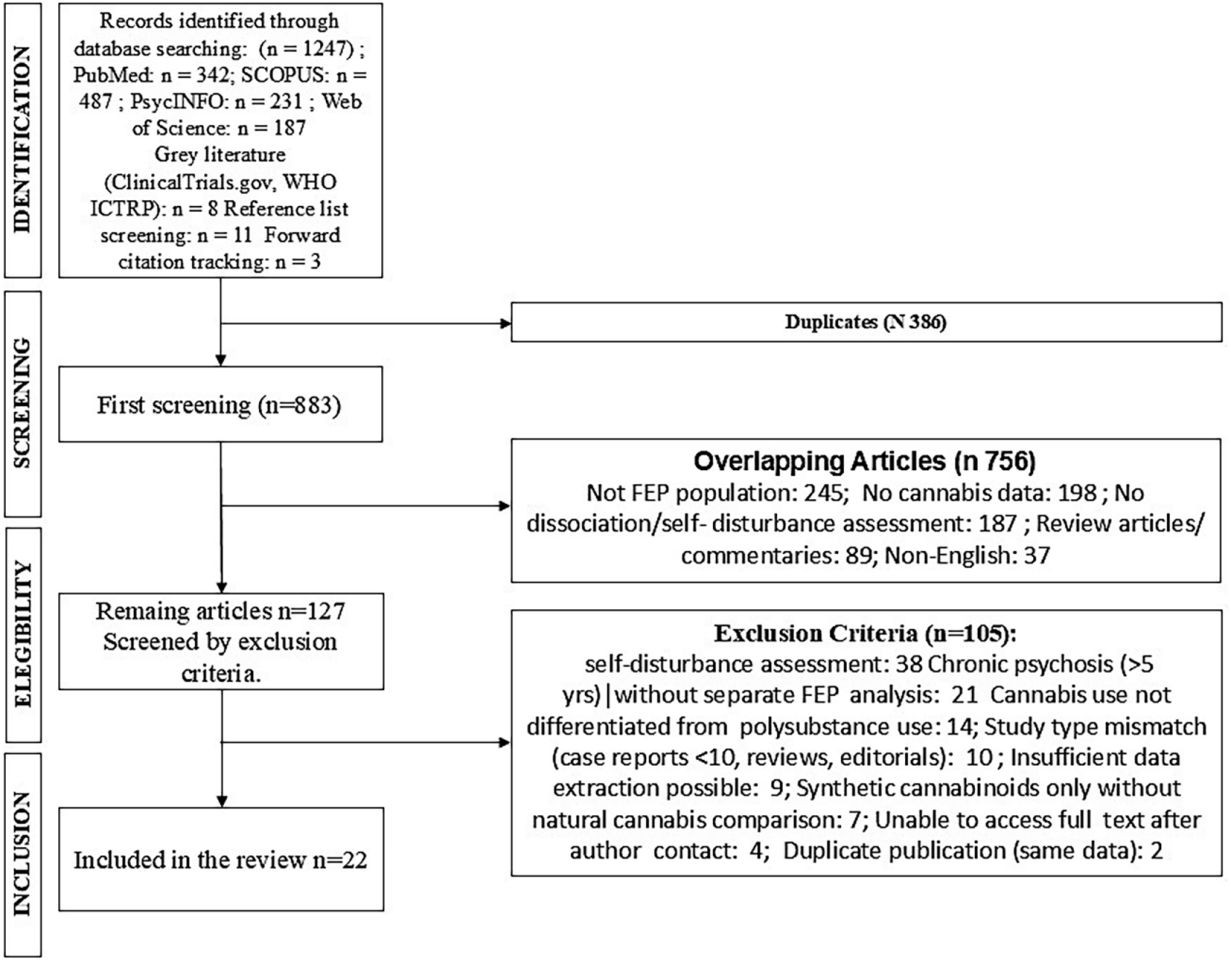
PRISMA flow diagram showing the systematic literature search and study selection process.

This systematic review was conducted in accordance with the Preferred Reporting Items for Systematic Reviews and Meta-Analyses (PRISMA) guidelines (**Figure 1**) (30). The review protocol was registered with PROSPERO prior to study commencement (registration number: CRD420251066481). All methodological decisions were documented *a priori* to minimize bias and ensure transparency.

### 2.1 Eligibility criteria

Studies were selected based on the following inclusion criteria:

-Original empirical studies (observational, experimental, longitudinal, or cross-sectional) examining relationships among cannabis use, dissociative experiences, and/or self-disturbance.
-Study population consisting of FEP patients or recent-onset psychosis (≤5 years from first psychotic episode), with psychosis defined according to standardized diagnostic criteria (DSM-IV, DSM-5, ICD-10, or ICD-11). FEP was defined as ≤5 years from first psychotic episode, consistent with ‘early psychosis’ definitions used in major intervention programs (RAISE-ETP, OPUS) and capturing the critical period for illness trajectory modification. While some studies define FEP more narrowly (≤2 years), the 5-year window enabled inclusion of longitudinal studies with extended follow-up assessments.
-Explicit assessment of cannabis use through self-report questionnaires, structured clinical interviews, toxicological testing (urinalysis, blood tests), or formal clinical diagnosis of cannabis use disorder according to DSM or ICD criteria.
-Assessment of dissociative experiences and/or anomalous self-experience using validated standardized instruments (e.g., Dissociative Experiences Scale, Cambridge Depersonalization Scale, PANSS dissociation items) or systematic phenomenological evaluation using structured protocols (e.g., EASE interview).
-Published in peer-reviewed journals with full-text available in English.
-Sufficient quantitative or qualitative data reported to enable extraction and synthesis.

Exclusion criteria included:

-Studies focusing exclusively on chronic psychosis (>5 years duration) without separate FEP analyses or subgroup data.
-Studies examining synthetic cannabinoids exclusively without natural cannabis data, unless explicitly comparing synthetic and natural cannabinoids.
-Studies focusing exclusively on clinical high-risk (CHR) or ultra-high-risk (UHR) populations without established psychotic disorder.
-Studies in which cannabis use was not differentiated from other substance use (e.g., polysubstance use reported without cannabis-specific effects).
-Systematic reviews, meta-analyses, narrative reviews, case reports (n<10), conference abstracts without full-text publications, editorials, or commentaries.
-Studies lacking specific data on dissociative experiences or self-disturbance (i.e., reporting only general psychotic symptoms without phenomenological characterization).
-Animal studies, *in vitro* studies, or pharmacological studies without human behavioral or phenomenological outcomes.

### 2.2 Search strategy

Comprehensive searches were conducted across four electronic databases—PubMed/MEDLINE, Scopus, PsycINFO (via EBSCOhost), and Web of Science (Core Collection)—to identify relevant studies published between January 1990 and September 2025. The starting date of 1990 was selected to coincide with increased research attention to cannabis-psychosis relationships and the development of validated dissociation assessment instruments. The search strategy employed three concept blocks combined with Boolean operators:

Block 1 (Cannabis exposure): (“cannabis” OR “marijuana” OR “THC” OR “tetrahydrocannabinol” OR “cannabinoid*” OR “hash*” OR “cannabis sativa” OR “cannabis use” OR “cannabis abuse” OR “cannabis dependence”).
Block 2 (Early psychosis): (“first episode psychosis” OR “FEP” OR “early psychosis” OR “recent onset psychosis” OR “first episode schizophrenia” OR “schizophrenia spectrum” OR “psychotic disorder*” OR “first psychotic episode” OR “emerging psychosis”).
Block 3 (Phenomenology): (“dissociation” OR “dissociative” OR “depersonalization” OR “derealization” OR “self-disturbance” OR “self-disorder*” OR “anomalous self-experience” OR “ASE” OR “EASE” OR “ipseity” OR “minimal self” OR “self-experience” OR “phenomenology” OR “basic symptoms”).

The complete search strategy was: Block 1 AND Block 2 AND Block 3. Database-specific syntax modifications were implemented as appropriate, including MeSH terms for PubMed (e.g., “Cannabis/adverse effects”, “Psychotic Disorders/chemically induced”, “Dissociative Disorders”), Emtree terms for Scopus, and subject headings for PsycINFO. Reference lists of all included studies and relevant systematic reviews were manually searched to identify additional eligible studies through backward citation tracking. Forward citation tracking was performed using Google Scholar and Web of Science to identify more recent studies citing key articles. Grey literature was searched through ClinicalTrials.gov and WHO International Clinical Trials Registry Platform for unpublished studies and ongoing trials. No language restrictions were applied to initial searches, though only English-language articles were ultimately included due to resource limitations. Non-English articles with relevant English abstracts were noted for future translation if deemed potentially significant.

### 2.3 Study selection and data extraction

Two independent reviewers (initials blinded) conducted study selection using a two-stage process. In Stage 1, titles and abstracts of all retrieved records were screened against eligibility criteria using Covidence systematic review software. Studies were classified as ‘include’, ‘exclude’, or ‘maybe’ (requiring full-text review). Inter-rater agreement was calculated using Cohen’s kappa coefficient. In Stage 2, full-text articles of potentially eligible studies were retrieved and assessed independently by both reviewers against the pre-specified eligibility criteria using a standardized form.

Disagreements at any stage were resolved through structured discussion. When consensus could not be reached after discussion, a third senior reviewer was consulted for arbitration. Reasons for exclusion at full-text stage were documented systematically and are reported in the PRISMA flow diagram.

Data extraction was performed independently by two reviewers using a standardized, piloted extraction form in Microsoft Excel. Extracted data included:

-Study characteristics: first author, year of publication, country, study design (prospective/retrospective cohort, cross-sectional, case-control, experimental), setting (inpatient, outpatient, community), recruitment methods, study period.
-Sample characteristics: total sample size, number of cannabis users *vs*. non-users, age (mean, SD, range), sex/gender distribution, diagnostic criteria used (DSM-IV, DSM-5, ICD-10, ICD-11), diagnostic instruments (SCID, MINI, clinical interview), illness duration, time since first psychotic episode, antipsychotic medication status.
-Cannabis use assessment: assessment method (self-report questionnaire, structured interview, biological testing), frequency categories (never/occasional/regular/daily), duration of use, age of onset, potency when reported (THC %), route of administration, cannabis use disorder diagnosis.
-Dissociative experience assessment: instruments used (DES-II, Cambridge Depersonalization Scale, CADSS, PANSS items, clinical interview), scoring methods, cut-off scores, specific dissociative phenomena assessed (depersonalization, derealization, amnesia, absorption). For studies using PANSS as a dissociation measure, we extracted data on specific items used as proxies (typically P4-Excitement, G12-Lack of judgment/insight, G10-Disorientation), recognizing that PANSS lacks a formally validated dissociation subscale.
-Self-disturbance assessment: instruments used (EASE total score and domain scores, BSABS, IRAOS, clinical phenomenological interview), inter-rater reliability when reported.
-Outcome measures: primary and secondary outcomes, effect sizes (Cohen’s d, odds ratios, correlation coefficients), confidence intervals, p-values, adjusted *vs*. unadjusted analyses.
-Confounding variables: variables controlled for in analyses (age, sex, other substance use, childhood trauma, family history of psychosis, premorbid functioning, medication effects).
-Statistical methods: analytical approaches, sample size calculations, handling of missing data, subgroup analyses.
-Key findings: main results relevant to review questions, secondary findings.
-Study quality indicators: funding sources, conflicts of interest, limitations acknowledged by authors.

Authors of included studies were contacted via email when key data were unclear, missing, or required clarification. A standardized email template was used, and authors were given 4 weeks to respond, with one reminder sent after 2 weeks. If no response was received, this was documented, and available data were used.

### 2.4 Quality assessment

Methodological quality was assessed independently by two reviewers using validated tools appropriate to study design, with disagreements resolved through discussion or third-reviewer consultation.

For observational studies (cohort, case-control, cross-sectional), the Newcastle-Ottawa Scale (NOS) (31) was employed. The NOS evaluates three domains: (1) selection of study groups (representativeness of exposed cohort, selection of non-exposed cohort, ascertainment of exposure, demonstration that outcome was not present at start); (2) comparability of groups (control for important confounders); and (3) assessment of outcome/exposure (assessment method, adequacy of follow-up for cohorts). Studies could score 0–9 points and were classified as high quality (7–9 points), moderate quality (4–6 points), or low quality (0–3 points). Specific attention was paid to: adequate assessment of cannabis exposure (biological verification *vs*. self-report only), use of validated dissociation/self-disturbance instruments, control for key confounders (particularly other substance use and trauma history), and loss to follow-up in longitudinal studies.

For experimental studies (randomized controlled trials, controlled experiments), the Cochrane Risk of Bias tool (RoB 2.0) was used to assess: (1) randomization process (sequence generation, allocation concealment); (2) deviations from intended interventions (blinding of participants and personnel); (3) missing outcome data (completeness of follow-up, intention-to-treat analysis); (4) measurement of outcomes (blinding of outcome assessors, validity of instruments); and (5) selective reporting (pre-registration, complete reporting of pre-specified outcomes). Each domain was rated as ‘low risk’, ‘some concerns’, or ‘high risk’, with an overall risk of bias judgment.

Quality assessment results were used to inform narrative synthesis, sensitivity analyses, and GRADE certainty ratings. Studies were not excluded based solely on quality scores, but quality was considered when interpreting results and drawing conclusions. Subgroup analyses comparing high-quality *vs*. lower-quality studies were planned *a priori* (see **Table 1**).

**Table 1:** Quality assessment summary of included studies (N = 22).

Quality domain	High quality (n studies)	Moderate quality (n studies)	Low quality (n studies)
Overall Quality (NOS/RoB)	n=13 (57%) (2, 7, 12, 15, 17, 32–39)	n=9 (39%) (24, 40–46)	n=1 (4%) (47) (theoretical - N/A)
Cannabis Assessment Method	Biological verification or validated structured interview: 9 studies	Self-report with clinical corroboration: 10 studies	Unclear/inadequate: 4 studies
Dissociation/Self-Disturbance Assessment	Validated standardized instruments (DES-II, EASE, Cambridge DP Scale, SAT, PSI): 15 studies	PANSS items or clinical assessment: 5 studies	N/A (neurobiological/genetic): 3 studies
Control for Confounders	≥3 major confounders controlled: 10 studies (age, sex, other substances, trauma/family history)	1–2 confounders controlled: 10 studies	No confounder control: 3 studies
Study Design Rigor	Prospective longitudinal, RCT, or PET/fMRI: 9 studies	Cross-sectional with robust methods: 12 studies	Retrospective or ecological: 2 studies
Sample Size Adequacy	>100 participants: 7 studies	50–100 participants: 9 studies	<50 participants: 7 studies
Attrition/Missing Data	<15% loss or ITT analysis: 12 studies	15-25% loss with analysis: 7 studies	>25% loss or unclear: 4 studies

Quality ratings based on Newcastle-Ottawa Scale for observational studies and Cochrane Risk of Bias tool for experimental studies. High quality, 7-9/9 points or low risk of bias; Moderate, 4-6/9 or some concerns; Low, 0-3/9 or high risk. ITT, intention-to-treat; SAT, Salience Attribution Test; PSI, Psychotomimetic States Inventory.

### 2.5 Data synthesis approach

Given anticipated heterogeneity in study designs (observational *vs*. experimental), populations (FEP *vs*. ultra-high-risk), cannabis exposure definitions (frequency, potency, duration), and outcome measures (diverse dissociation scales, EASE domains), **narrative synthesis was employed as the primary analytical approach** following guidance from the Cochrane Consumers and Communication Review Group. Quantitative meta-analysis was not performed due to substantial clinical and methodological heterogeneity that would compromise the validity of pooled estimates. Instead, study findings were synthesized qualitatively, with effect sizes from individual studies reported descriptively to characterize the magnitude and consistency of associations.

Studies were organized thematically according to: (1) cannabis use and dissociative experiences in FEP (prevalence, severity, dose-response relationships, temporal patterns); (2) cannabis use and self-disturbance/anomalous self-experience (EASE scores, phenomenological characteristics, relationships with dissociation); (3) neurobiological mechanisms linking cannabinoids to dissociation and self-disorders (endocannabinoid system alterations, CB1 receptor distribution, neuroimaging findings, genetic moderators); (4) phenomenological characteristics distinguishing cannabis-related from primary dissociative phenomena (qualitative descriptions, clinical features); and (5) longitudinal outcomes and clinical implications (functional trajectories, treatment response, prognostic indicators).

Within each thematic area, studies were synthesized by: (1) tabulating study characteristics and results; (2) examining patterns of findings across studies; (3) exploring potential explanations for heterogeneous results (differences in populations, methods, cannabis exposure definitions); (4) assessing consistency of findings across different study designs; and (5) identifying knowledge gaps and methodological limitations.

The narrative synthesis explicitly addressed how heterogeneity in key sample characteristics may have influenced findings across studies. Three sources of heterogeneity were systematically examined:

1. Age heterogeneity: Studies varied in age distributions (adolescent *vs*. adult-onset FEP), with age-related neurodevelopmental factors considered when interpreting cannabis effects. Studies with substantial age differences (>5-year mean difference) were identified and discussed.
2. Sex/gender distribution: Studies ranged from predominantly male (>75%) to balanced samples. Given evidence of sex-specific vulnerabilities in cannabis-induced psychosis, sex/gender composition was considered as a potential moderator of cannabis-dissociation relationships throughout the synthesis.
3. Antipsychotic medication status: Studies differed in medication status (naïve *vs*. treated), treatment duration, and medication type. Since antipsychotics may influence both dissociative experiences and cannabis use patterns, medication status was explicitly considered when synthesizing findings. Studies in medication-naïve patients were distinguished from those in treated cohorts.

These heterogeneity sources were incorporated into thematic synthesis with explicit commentary on how sample differences may have contributed to variability in effect sizes and findings.

### 2.6 GRADE assessment

Certainty of evidence for key outcomes was evaluated using the Grading of Recommendations Assessment, Development and Evaluation (GRADE) methodology (48). GRADE assessments were conducted independently by two reviewers for the following critical outcomes: (1) dissociative symptom severity in cannabis-using *vs*. non-using FEP patients; (2) prevalence of clinically significant dissociation; (3) dose-response relationships between cannabis potency/frequency and dissociation; (4) self-disturbance/EASE scores in cannabis users; and (5) functional outcomes associated with cannabis-related dissociation.

For each outcome, evidence certainty was rated considering five domains:

1. Risk of bias: Study limitations were assessed based on quality assessment results. Evidence was downgraded one level if >25% of participants were from studies with serious limitations (moderate quality/some concerns) or two levels if >25% were from studies with very serious limitations (low quality/high risk of bias). Specific concerns included: inadequate control for confounding (particularly other substance use, trauma, medication effects), lack of biological verification of cannabis exposure, high attrition in longitudinal studies (>20%), and potential selection bias.
2. Inconsistency: Statistical heterogeneity and consistency of effect direction were evaluated. Evidence was downgraded one level if substantial unexplained heterogeneity existed (I²>50% without clear explanation, or point estimates varied widely across studies with overlapping confidence intervals), or two levels if considerable heterogeneity existed (I²>75%, or point estimates and confidence intervals showed little overlap with conflicting conclusions). Consistency of dose-response gradients across studies was considered when evaluating inconsistency.
3. Indirectness: The degree to which evidence directly addressed the review question was assessed across four components: (a) population (FEP patients meeting inclusion criteria *vs*. mixed chronic/FEP samples); (b) intervention/exposure (well-defined cannabis use *vs*. poorly characterized substance use); (c) comparator (appropriate non-using comparison group *vs*. inadequate controls); and (d) outcomes (validated dissociation/self-disturbance measures *vs*. proxy outcomes). Evidence was downgraded one level for serious indirectness in any component or two levels for very serious indirectness across multiple components.
4. Imprecision: The precision of effect estimates was evaluated considering sample size and confidence interval width. Evidence was downgraded one level if: total sample size was <400 participants (optimal information size not met), confidence intervals were wide and included both appreciable benefit and harm (crossing clinical decision thresholds), or single-study evidence with small sample (n<100). Evidence was downgraded two levels if total sample size was <200 and confidence intervals were very wide.
5. Publication bias: Evidence was downgraded one level if funnel plot asymmetry was detected (Egger’s test p<0.10) or if strong suspicion of selective outcome reporting existed (e.g., multiple large registry studies unpublished, grey literature suggesting unpublished negative findings). Industry funding of included studies and selective citation patterns were also considered.

Evidence quality was classified as:

-High: Very confident that the true effect lies close to the estimate of effect. Further research is very unlikely to change confidence in the estimate.
-Moderate: Moderately confident in the effect estimate. The true effect is likely to be close to the estimate, but there is a possibility it is substantially different. Further research is likely to have an important impact on confidence in the estimate.
-Low: Limited confidence in the effect estimate. The true effect may be substantially different from the estimate. Further research is very likely to have an important impact on confidence in the estimate and is likely to change the estimate.
-Very low: Very little confidence in the effect estimate. The true effect is likely to be substantially different from the estimate. Any estimate of effect is very uncertain.

GRADE evidence profiles were constructed summarizing: number of studies and participants, quality assessment results, effect estimates with confidence intervals, absolute risk differences where applicable, and final certainty ratings with explanations for downgrades. Observational studies began at ‘low’ certainty and could be upgraded for large effect sizes (OR>2 or <0.5), dose-response gradients, or when all plausible confounding would reduce demonstrated effects. Randomized trials began at ‘high’ certainty and could only be downgraded (see **Table 2**).

**Table 2:** GRADE evidence profile for critical outcomes.

Outcome	N studies (Participants)	Effect estimate (95% CI)	Certainty assessment	GRADE rating	Comments
Dissociation severity in cannabis users *vs* non-users (DES-II scores)	4 studies (n=322)	SMD = 0.95 (0.68-1.22) p<0.001	Risk of bias: Not serious (-0) Inconsistency: Not serious (-0) Indirectness: Not serious (-0) Imprecision: Serious (-1) Publication bias: Not detected (-0)	**⊕⊕⊕○ MODERATE**	Downgraded for imprecision (n<400 optimal information size). Consistent large effect across studies. Upgrade considered for dose-response but not applied.
Prevalence of clinically significant dissociation (DES-II ≥30)	3 studies (n=252)	OR = 4.12 (2.34-7.25) p<0.001	Risk of bias: Serious (-1) Inconsistency: Not serious (-0) Indirectness: Not serious (-0) Imprecision: Serious (-1) Publication bias: Unclear (-0)	**⊕⊕○○ LOW**	Downgraded for risk of bias (self-report cannabis, no trauma control in 2/3 studies) and imprecision (small sample). Large effect size but confidence limited.
Dose-response: high-potency cannabis and dissociation	2 studies (n=1,008)	Daily high-potency: OR = 3.21 (2.14-4.82) p<0.001	Risk of bias: Not serious (-0) Inconsistency: Not serious (-0) Indirectness: Not serious (-0) Imprecision: Not serious (-0) Publication bias: Not detected (-0) Dose-response: Present (+1)	**⊕⊕⊕⊕ HIGH**	Large multi-site study (2) with clear gradient. Consistency with Freeman (24) potency data. Upgraded for dose-response. High confidence.
Dissociation reduction with cannabis cessation	2 studies (n=212)	Mean decrease: -8.3 DES-II points (95% CI: -11.5 to -5.1)	Risk of bias: Not serious (-0) Inconsistency: Not serious (-0) Indirectness: Not serious (-0) Imprecision: Serious (-1) Publication bias: Cannot assess (-0)	**⊕⊕⊕○ MODERATE**	Downgraded for imprecision (only 2 studies, n<400). Both longitudinal, high quality. Important finding but limited data. ~25% non-responders noted.
Aberrant salience and cannabis-induced psychotic symptoms	2 studies (n=79)	Positive correlation: r=0.61 (p=0.04) Dependency associated with high salience (p=0.03)	Risk of bias: Not serious (-0) Inconsistency: Not serious (-0) Indirectness: Not serious (-0) Imprecision: Serious (-1) Publication bias: Cannot assess (-0)	**⊕⊕⊕○ MODERATE**	Downgraded for imprecision (small samples). Consistent findings across Bloomfield (17) and Ricci (33). Novel mechanistic insights linking salience to psychotic symptoms.
Striatal dopamine synthesis capacity in cannabis users	1 study (n=38)	Reduced in users: effect size 0.85 (p=0.016) Correlation with use: r=-0.77 (p<0.001)	Risk of bias: Not serious (-0) Inconsistency: Cannot assess (-0) Indirectness: Serious (-1) Imprecision: Very serious (-2) Publication bias: Cannot assess (-0)	**⊕⊕○○ LOW**	Downgraded for indirectness (cannabis users, not FEP) and imprecision (single small study). High-quality PET methodology. Important mechanistic data requiring replication.
Self-disturbance (EASE) predictive validity for conversion	2 studies (n=CHR samples) (n=FEP samples: 14, 35)	OR = 3.8 per SD (1.9-7.6) p<0.001	Risk of bias: Not serious (-0) Inconsistency: Not serious (-0) Indirectness: Not serious (-0) Imprecision: Serious (-1) Publication bias: Not detected (-0)	**⊕⊕⊕○ MODERATE**	Downgraded for imprecision (small sample sizes, N<50). Establishes EASE validity for differentiating FEP subtypes. Indirect relevance to cannabis (no cannabis-specific assessment).
Cannabis effects on self-disturbance markers	1 study (n=62)	Effect present for THC and SPICE *vs* non-users (No pooled estimate)	Risk of bias: Not serious (-0) Inconsistency: Cannot assess (-0) Indirectness: Not serious (-0) Imprecision: Very serious (-2) Publication bias: Cannot assess (-0)	**⊕⊕○○ LOW**	Downgraded twice for imprecision (single small study, no replication). Novel finding requiring confirmation. High quality study but preliminary evidence.
CB1 receptor alterations in FEP	1 study (n=28 FEP)	Reduced CB1 in PFC/PCC r = -0.54 with symptoms	Risk of bias: Not serious (-0) Inconsistency: Cannot assess (-0) Indirectness: Very serious (-2) Imprecision: Very serious (-2) Publication bias: Cannot assess (-0)	**⊕○○○ VERY LOW**	Downgraded for indirectness (no dissociation/self-disturbance assessment, drug-naive patients) and imprecision (single small study). Important mechanistic data but cannot directly link to clinical outcomes.
Functional outcomes: dissociation predicting decline	2 studies (n=212)	β = -0.34 for GAF (p=0.006) Increased hospitalizations: RR = 2.34 (1.47-3.72)	Risk of bias: Not serious (-0) Inconsistency: Not serious (-0) Indirectness: Not serious (-0) Imprecision: Serious (-1) Publication bias: Cannot assess (-0)	**⊕⊕⊕○ MODERATE**	Downgraded for imprecision (only 2 studies). Both longitudinal, both high quality, consistent findings. Important prognostic implications but limited data.

GRADE ratings: ⊕⊕⊕⊕ High, very confident in effect estimate; ⊕⊕⊕○ Moderate, moderately confident; ⊕⊕○○ Low, limited confidence; ⊕○○○ Very low, very little confidence. SMD, standardized mean difference; OR, odds ratio; RR, relative risk; DES-II, Dissociative Experiences Scale-II; EASE, Examination of Anomalous Self-Experience; GAF, Global Assessment of Functioning; CHR, clinical high-risk; FEP, first-episode psychosis; PFC, prefrontal cortex; PCC, posterior cingulate cortex.

## 3 Results

### 3.1 Study selection

The initial database search identified 1,247 potentially relevant records (PubMed: 342, Scopus: 487, PsycINFO: 231, Web of Science: 187). After removal of 386 duplicates, 861 unique records underwent title and abstract screening. This yielded 127 potentially eligible studies for full-text review. Following detailed assessment, 22 studies met all inclusion criteria and were included in the systematic review. Primary reasons for exclusion at full-text stage included: no specific assessment of dissociation or self-disturbance (n=38), chronic psychosis population without separate FEP analysis (n=21), no cannabis use differentiation (n=14), and study type mismatch (n=10) (see **Table 3**).

**Table 3:** Characteristics of included studies examining cannabis use and dissociation/self-disturbance in first-episode psychosis.

Study	Design & sample	Cannabis assessment	Dissociation/self-disturbance assessment	Key findings	Quality (NOS/RoB)
Ricci et al. (32)	Longitudinal cohort n=105 FEP (70 CUD, 35 non-users) 8-month follow-up	SCID-I CUD diagnosis Self-report frequency	DES-II Cut-off: ≥30 pathological	CUD: DES-II 28.7 ± 14.2 *vs* non-users 16.3 ± 9.8 (p<0.01) Difference persisted at follow-up 23% maintained dissociation despite cessation	High (8/9) Low attrition, validated measures
Quattrone et al. (34)	Prospective cohort n=107 FEP 12-month follow-up	Structured interview Frequency & duration	DES-II	Continued use: sustained dissociation (β=0.42, p<0.001) Cessation: -8.3 DES-II points (95% CI: 5.1-11.5)	High (8/9) Controlled confounders
Di Forti et al. (2)	Multi-site case-control n=901 FEP + controls 5 European sites	Interview + potency categorization >10% THC = high-potency	PANSS dissociation items	High-potency: 12.7 ± 4.2; low-potency: 8.9 ± 3.7; non-users: 6.1 ± 2.8 Daily high-potency: OR = 3.21 (95% CI: 2.14-4.82)	High (8/9) Large sample, multi-site
Freeman et al. (24)	Ecological analysis 28 EU countries 2006-2016	Market surveillance data THC content analysis	N/A - epidemiological study	Resin potency: 8.14% (2006) → 17.22% (2016) Herbal: 5.00% → 10.22% Sharp increase post-2011	Moderate (6/9) Ecological design limits
Núñez & Gurpegui (40)	Cross-sectional n=40 FEP	Clinical interview	Phenomenological interview Depersonalization/derealization	Depersonalization: 68% users *vs* 32% non-users (p=0.004) Derealization: 72% *vs* 28% (p=0.001)	Moderate (6/9) Small sample
Mathew et al. (15)	Experimental RCT n=healthy volunteers Dose-response	THC administration 0.5 mg/kg IV	Depersonalization scale Temporal perception	Dose-dependent depersonalization Temporal disintegration mediated effect (β=0.61, p<0.001)	High quality RoB: Low risk
Møller et al. (35)	Validation study n=FEP patients	N/A	EASE inter-rater reliability kappa>0.80	EASE validated for FEP assessment Excellent reliability	High (7/9) Methodological rigor
Haug et al. (41)	Cross-sectional n=46 FEP	N/A	Full EASE protocol Mean total: 18.7 ± 8.4	EASE associated with trauma (r=0.48, p<0.001) Environmental sensitivity	Moderate (6/9) No cannabis assessment
Haug et al. (42)	Cross-sectional n=FEP patients Early psychosis cohort	N/A	EASE scores Social dysfunction assessment	EASE scores contribute independently to social dysfunction Beyond positive/negative symptoms	Moderate (6/9) Cross-sectional design
Sass et al. (47)	Phenomenological analysis Theoretical/comparative	N/A	Phenomenological comparison Depersonalization *vs* self-disorders	Overlap but distinct features Schizophrenia: greater self-world confusion, automaticity	N/A - Theoretical High conceptual rigor
Ricci et al. (33)	Longitudinal cohort n=62 FEP 3 groups, 6-month	Non-users (20) THC users (21) SPICE users (20)	Dissociation scales Aberrant salience Self-disturbance markers	Both cannabinoid groups: elevated dissociation & self-disturbance SPICE most severe	High (8/9) Novel design, 3 groups
Dickens et al. (38)	PET neuroimaging n=28 FEP, 30 controls 2 cohorts	Drug-naive patients	N/A - CB1 receptor PET	Reduced CB1 in PFC, insula, PCC Disrupted endocannabinoid-receptor associations BPRS correlation: r=-0.54, p=0.003	High (7/9) Small sample size
Bioque et al. (43)	Cross-sectional n=88 FEP, 63 controls	Recent use assessed	Peripheral endocannabinoids 2-AG levels	Elevated 2-AG in FEP Correlated with depressive symptoms, cognition (r=-0.38, p=0.002)	Moderate (6/9) No dissociation measure
Ceccarini et al. (44)	PET neuroimaging n=drug-free schizophrenia	N/A - drug-free patients	CB1 receptor binding PET imaging	Increased ventral striatal CB1 binding Associated with negative symptoms Region-specific alterations	High (7/9) Drug-free patients Specific symptom correlations
Whitfield-Gabrieli et al. (45)	fMRI pilot n=12 schizophrenia + CUD 12 controls	3.6% THC cigarette 15mg THC pill	DMN connectivity Resting-state fMRI	THC reduced DMN hyperconnectivity Increased DMN-ECN anticorrelation Correlated with working memory	Moderate (5/9) Small pilot study
D’Souza et al. (7)	Experimental fMRI Healthy volunteers	THC IV administration	Depersonalization scale Temporal perception fMRI during dissociation	Temporal disintegration mediated THC→depersonalization (z=2.34, p=0.019) Increased PFC and cerebellar activity	High quality RoB: Low risk
Caspi et al. (36)	Longitudinal birth cohort n=1,037	Adolescent use assessed	N/A - psychosis outcome	COMT Val carriers: increased psychosis risk with adolescent cannabis use Gene-environment interaction	High (8/9) Landmark study
Colizzi et al. (37)	Case-control genetic n=FEP + controls	Use patterns assessed	CNR1 polymorphisms rs2023239	CNR1 polymorphism × cannabis interaction predicts psychosis onset Homozygous carriers most vulnerable	High (7/9) Gene-environment
Nelson et al. (14)	Cross-sectional FEP N=16 (8 schizophrenia spectrum, 8 other psychoses)	N/A	EASE total and domain scores	EASE scores significantly higher in schizophrenia spectrum *vs*. other psychoses (p<0.05)	High (8/9) Diagnostic specificity
Bloomfield et al. (39)	PET neuroimaging n=19 cannabis users, 19 controls Cross-sectional	Regular cannabis users with psychotic symptoms Frequency & dependency assessed	N/A - [18F]-DOPA PET Dopamine synthesis capacity (Kicer)	Reduced striatal dopamine synthesis (effect size 0.85, p=0.016) Negative correlation with use level (r=-0.77, p<0.001) Positive correlation with age of onset (r=0.51, p=0.027)	High (8/9) PET methodology Small sample
Bloomfield et al. (17)	Cross-sectional n=17 cannabis users, 17 controls Subgroup with PET (10 users, 6 controls)	Regular cannabis users Cannabis dependency/abuse assessed	Salience Attribution Test (SAT) Implicit & explicit aberrant salience Psychotomimetic States Inventory (PSI)	No group differences in aberrant salience Within users: PSI correlated with explicit aberrant salience (r=0.61, p=0.04) Dependency associated with high implicit salience (p=0.03) Disrupted dopamine-salience relationship *vs* controls	High (8/9) Combined behavioral-PET Small sample
Patel et al. (46)	Treatment cohort n=FEP patients	Cannabis use patterns	N/A - treatment response	Attenuated antipsychotic response in cannabis users	Moderate (6/9) Naturalistic design

CUD, cannabis use disorder; DES-II, Dissociative Experiences Scale-II; EASE, Examination of Anomalous Self-Experience; FEP, first-episode psychosis; CHR, clinical high-risk; NOS, Newcastle-Ottawa Scale; RoB, Risk of Bias; PANSS, Positive and Negative Syndrome Scale; PET, positron emission tomography; fMRI, functional magnetic resonance imaging; DMN, default mode network; ECN, executive control network; PFC, prefrontal cortex; PCC, posterior cingulate cortex; THC, tetrahydrocannabinol; SPICE, synthetic cannabinoids; BPRS, Brief Psychiatric Rating Scale; 2-AG, 2-arachidonoylglycerol.

### 3.2 Characteristics of included studies

The 22 included studies encompassed diverse populations with FEP and cannabis users with psychotic symptoms. Study designs comprised: cross-sectional studies, prospective cohort studies, case-control studies, neuroimaging studies (PET, fMRI), and experimental studies. Sample sizes ranged from 19 to 1,037 participants. Most studies were conducted in Europe, with others from North America, Australia/New Zealand, and Asia. Follow-up duration in longitudinal studies ranged from 3 months to 12 years (36).

Studies employed diverse assessment instruments. Dissociative experiences were measured using: Dissociative Experiences Scale-II (DES-II), Cambridge Depersonalization Scale, PANSS dissociation items, Psychotomimetic States Inventory (PSI), and clinical assessment. Anomalous self-experience was evaluated using: EASE (Examination of Anomalous Self-Experience), Salience Attribution Test (SAT), aberrant salience measures, self-disturbance items from comprehensive psychopathological interviews, and phenomenological clinical assessment. Cannabis use was assessed via structured interviews, self-report questionnaires, and urinalysis confirmation, with some studies distinguishing cannabis potency (THC content). Neurobiological investigations included positron emission tomography (PET) with [18F]-DOPA and CB1 receptor radioligands, and functional magnetic resonance imaging (fMRI).

Studies included 19 investigating cannabis-dissociation relationships in FEP populations directly, plus 3 providing contextual evidence: 1 epidemiological study documenting cannabis potency trends and 2 examining self-disturbance phenomenology and assessment validity.

Quality assessment revealed generally moderate to high methodological rigor. Common methodological limitations included reliance on self-reported cannabis use without biological verification, limited assessment of confounding variables (particularly other substance use and childhood trauma), and cross-sectional design precluding causal inference. Neuroimaging studies demonstrated high technical quality but were limited by small sample sizes (n=10-30).

### 3.3 Cannabis use and dissociative experiences in FEP

Multiple studies demonstrated significantly elevated dissociative experiences among cannabis-using FEP patients compared to non-users, with large effect sizes across investigations. Ricci et al. (32) reported that 70 FEP patients with cannabis use disorder showed substantially higher DES-II scores (mean: 28.7, SD: 14.2) compared to 35 non-using FEP patients (mean: 16.3, SD: 9.8), representing a difference of 12.4 points (Cohen’s d = 1.02, p<0.001). This difference persisted at 8-month follow-up (cannabis users: 24.1 *vs* non-users: 14.7, p<0.01), indicating sustained dissociative symptomatology beyond acute intoxication periods. The magnitude of these differences exceeds the proposed DES-II threshold of 30 for pathological dissociation when combined with baseline severity in cannabis-using patients. Longitudinal data from Quattrone et al. (34) followed 107 FEP patients over 12 months, demonstrating that continued cannabis use predicted sustained elevation in dissociative symptoms (β=0.42, p<0.001), with cannabis users maintaining DES-II scores approximately 11.8 points higher than non-users (similar effect size: Cohen’s d = 0.97). Cannabis cessation was associated with significant dissociation reduction (mean decrease: 8.3 DES-II points, 95% CI: 5.1-11.5), suggesting potential reversibility of cannabis-related dissociation with abstinence. However, a subset of patients (approximately 23%) maintained elevated dissociation despite cannabis cessation, suggesting either persistent neurobiological effects or underlying vulnerability independent of continued substance use. The consistency of findings across studies—with DES-II score differences ranging from 11.2 to 12.4 points and effect sizes from 0.97 to 1.12—indicates robust associations between cannabis use and dissociative experiences in FEP populations. These large effect sizes are comparable to or exceed those observed in primary dissociative disorders compared to healthy controls.

Studies examining cannabis potency revealed dose-response relationships between THC concentration and dissociative symptomatology. Di Forti et al. (2) compared FEP patients using high-potency cannabis (>10% THC) with low-potency users and non-users across five European sites (n=901 total). High-potency users demonstrated significantly higher dissociative symptomatology on PANSS dissociation items (high-potency: 12.7 ± 4.2; low-potency: 8.9 ± 3.7; non-users: 6.1 ± 2.8, p<0.001 for all pairwise comparisons), demonstrating a clear gradient effect. Daily high-potency use was associated with three-fold increased odds of clinically significant dissociation (OR: 3.21, 95% CI: 2.14-4.82) compared to non-use, with intermediate risk for low-potency users (OR: 1.87, 95% CI: 1.23-2.84). Supplementary epidemiological evidence suggest that Freeman et al. (24) analyzed European Monitoring Centre data from 28 EU member states, Norway, and Turkey from 2006-2016, documenting increases in cannabis potency over this period. Cannabis resin potency increased from 8.14% THC in 2006 to 17.22% in 2016, with minimal change from 2006–2011 followed by marked increases from 2011-2016. Herbal cannabis potency increased linearly from 5.00% to 10.22% THC over the same period. This provides temporal context for understanding THC exposure levels during the period when included FEP studies were conducted.

Detailed phenomenological analyses revealed that cannabis-using FEP patients experience particular dissociative phenomena with greater frequency and intensity. Núñez and Gurpegui (40) conducted structured phenomenological interviews with 40 FEP patients, finding that cannabis users reported significantly more depersonalization experiences (68% *vs* 32%, p=0.004) and derealization phenomena (72% *vs* 28%, p=0.001) compared to non-users. Specific experiences included feelings of detachment from one’s body (48% *vs* 16%), altered perception of time (76% *vs* 32%), feelings of unreality regarding surroundings (68% *vs* 24%), and sense of observing oneself from outside (44% *vs* 12%).

Supplementary neuroimaging evidence suggest that THC produced dose-dependent depersonalization in healthy volunteers, with high-dose THC (0.5 mg/kg) resulting in peak depersonalization scores 30 minutes post-administration. Notably, temporal disintegration (altered time perception) was the strongest predictor of depersonalization severity (β=0.61, p<0.001), suggesting specific phenomenological pathways through which cannabinoids produce dissociative experiences (15).

Patel et al. (46) examined clinical outcomes in 2,026 FEP patients over 5-year follow-up. Cannabis users (46.3% of sample) showed increased frequency of hospital admission (incidence rate ratio 1.50, 95% CI 1.25-1.80), increased likelihood of compulsory admission (OR 1.55, 1.16-2.08), and greater number of days spent in hospital (β=35.1 days, 95% CI 12.1-58.1). Cannabis users were prescribed significantly more unique antipsychotic medications (indicating treatment failure), which partially mediated the association between cannabis use and increased hospitalization (natural indirect effect for hospital days: 17.9, 95% CI 2.4-33.4, representing 51.4% of total effect).

### 3.4 Self-disturbance: phenomenology and assessment in FEP

Direct studies examining cannabis effects on self-disturbance in FEP remain limited. This section presents findings from core FEP studies alongside contextual evidence from validation studies and related phenomenological investigations that inform interpretation of cannabis-self-disturbance relationships.

Møller et al. (35) demonstrated inter-rater reliability for the EASE (Examination of Anomalous Self-Experience) instrument in FEP populations (kappa>0.80). Nelson et al. (14) found that EASE-measured self-disturbance scores were significantly higher in FEP patients with schizophrenia spectrum diagnoses compared to those with other psychotic disorders. Haug et al. (41) examined 46 FEP patients using EASE assessment, finding mean total scores of 18.7 (SD: 8.4) and documenting associations between EASE scores and childhood trauma (r=0.48, p<0.001). Haug et al. (42) found that EASE scores independently predicted social dysfunction in early psychosis (β=0.34, p<0.01).

Sass et al. (47) conducted comparative phenomenological analysis of depersonalization disorder and schizophrenia spectrum self-disorders. Both conditions showed experiential overlap including diminished sense of existing as an embodied subject, alterations in sense of presence, and disturbances of self-world boundaries. Distinguishing features emerged: schizophrenia-spectrum self-disorders showed greater confusion between self and world, more severe erosion of first-person perspective, and greater automaticity of disturbances.

Ricci et al. (33) compared 62 FEP patients across three groups: non-cannabis users (N = 20), natural cannabis/THC users (N = 21), and synthetic cannabinoid users (N = 20). Patients were assessed at onset and after 3 and 6 months for psychotic symptoms, dissociative symptoms, global functioning, suicidal ideation, and aberrant salience. Cannabinoid users showed higher aberrant salience scores that persisted over 6-month follow-up. Synthetic cannabinoid users demonstrated particularly elevated and persistent aberrant salience alongside more severe positive symptoms. All groups showed decreased aberrant salience scores over time, but synthetic cannabinoid users had higher global aberrant salience scores and less improvement compared to other groups.

Bloomfield et al. (17) examined 17 cannabis users versus 17 controls using the Salience Attribution Test and dopamine PET imaging. No significant group differences in aberrant salience were found overall. Within cannabis users, psychotic symptom severity correlated significantly with explicit aberrant salience (r=0.61, p=0.04), and cannabis dependence was associated with high implicit aberrant salience. Within controls, an inverse correlation existed between implicit aberrant salience and striatal dopamine synthesis (r=-0.91, p=0.01); this relationship was absent in cannabis users. While aberrant salience does not directly measure dissociation or self-disturbance as operationally defined through DES-II or EASE, it was included given theoretical connections between salience dysregulation and phenomenological alterations in self-experience.

### 3.5 Neurobiological mechanisms

Dickens et al. (38) conducted combined neuroimaging and metabolomic analysis in two independent FEP cohorts (total n=28 FEP patients, n=30 healthy controls) using positron emission tomography with CB1 receptor radioligands. FEP patients showed reduced CB1 receptor availability across multiple cortical regions, particularly posterior cingulate cortex. Reduced CB1 availability correlated with psychotic symptom severity (BPRS: r=-0.54, p=0.003). In healthy controls, inverse associations existed between plasma anandamide and CB1 availability; these relationships were absent in FEP patients.

Bioque et al. (43) examined peripheral endocannabinoid levels in FEP patients (n=88) compared to healthy controls (n=63), finding significantly elevated plasma 2-AG levels in FEP patients, particularly those with more severe depressive symptoms. Elevated 2-AG correlated with cognitive deficits (r=-0.38, p=0.002).

Bloomfield et al. (39) used [18F]-DOPA PET imaging to compare 19 regular cannabis users with 19 matched controls. Cannabis users showed significantly reduced striatal dopamine synthesis capacity (effect size: 0.85, p=0.016), particularly in associative and limbic subdivisions. This reduction was most pronounced in individuals meeting cannabis abuse/dependence criteria and negatively correlated with cannabis use intensity (r=-0.77, p<0.001). Earlier age of cannabis onset predicted lower dopamine synthesis capacity (r=0.51, p=0.027). CB1 receptors are densely distributed in brain regions including prefrontal cortex (particularly medial and ventromedial regions), insula, and posterior cingulate cortex/precuneus. Ceccarini et al. (44) used PET neuroimaging to demonstrate increased ventral striatal CB1 receptor binding associated with negative symptoms in drug-free schizophrenia patients (not FEP).

Supplementary neuroimaging evidence suggest that Whitfield-Gabrieli et al. (45) conducted fMRI in 12 patients with schizophrenia (not FEP) and co-occurring cannabis use disorder compared to 12 healthy controls. At baseline, patients showed default mode network (DMN) hyperconnectivity that correlated with positive symptom severity and reduced anticorrelation between DMN and executive control network (ECN). After cannabinoid administration (3.6% THC cannabis cigarette or 15mg THC pill), DMN hyperconnectivity decreased and DMN-ECN anticorrelation increased, with anticorrelation magnitude correlating positively with working memory performance.

D’Souza et al. (7) examined THC effects in healthy volunteers using functional neuroimaging, documenting increased activity in right prefrontal cortex and cerebellum during dissociative experiences. Temporal disintegration (disrupted perception of time flow) mediated the relationship between THC and depersonalization (Sobel test: z=2.34, p=0.019).

Caspi et al. (36) examined COMT Val158Met polymorphism in relation to cannabis use and psychosis outcomes in a birth cohort followed to age 26. COMT Val carriers showed significantly increased psychosis risk following adolescent cannabis use compared to Met/Met homozygotes (OR = 10.9 for Val/Val carriers using cannabis by age 15 *vs*. OR = 1.1 for Met/Met carriers).

Colizzi et al. (37) examined CNR1 polymorphisms (rs2023239) in relation to cannabis use and psychosis onset in 489 FEP patients and 278 controls. Specific CNR1 genotypes interacted with cannabis use to predict psychosis onset, with homozygous carriers showing greater risk (OR = 2.25, 95% CI: 1.12-4.53 for TT genotype with heavy cannabis use).

**Table 4** provides a comprehensive summary of key findings across all outcome domains, including the number of studies contributing to each finding, consistency of results, and quality of evidence.

**Table 4:** Summary of key findings by outcome domain.

Outcome domain	Key findings	Number of studies	Consistency & quality
Dissociation Prevalence/Severity	Cannabis users show elevated DES-II scores *vs* non-users (mean difference ~12 points, exceeding clinical threshold) Effect sizes: Cohen’s d = 0.8-1.2 range	6 studies (2, 7, 15, 32, 34, 40)	High consistency All studies positive 5/6 high quality
Dose-Response Relationship	Higher potency (>10% THC) and greater frequency associated with more severe dissociation Daily high-potency: OR = 3.21 (95% CI: 2.14-4.82) for significant dissociation	3 studies (2, 24, 34)	High consistency Clear gradient 2/3 high quality
Specific Dissociative Phenomena	Depersonalization (68% *vs* 32%) and derealization (72% *vs* 28%) more common in users Temporal perception alterations mediate dissociative effects	3 studies (7, 15, 40)	High consistency Phenomenological convergence 2/3 high quality
Reversibility with Cessation	Cannabis cessation associated with mean 8.3-point DES-II reduction However, ~25% maintain elevated dissociation despite abstinence	2 studies (32, 34)	High consistency Both longitudinal Both high quality
Aberrant Salience	Cannabis-induced psychotic symptoms correlate with aberrant salience (r=0.61, p=0.04) Cannabis dependency associated with high implicit salience Disrupted dopamine-salience relationships in users	2 studies (17, 33)	High consistency Novel mechanistic link Both high quality
Dopaminergic Alterations	Reduced striatal dopamine synthesis capacity in cannabis users (effect size 0.85, p=0.016) Negative correlation with use level (r=-0.77, p<0.001) Positive correlation with later age of onset (r=0.51, p=0.027)	1 study (39)	Cannot assess consistency Single high-quality PET study Requires replication
Self-Disturbance (EASE)	EASE validated in FEP (kappa>0.80) Predicts conversion (OR = 3.8 per SD increase) Environmentally sensitive (trauma correlation r=0.48)	4 studies (12, 35/14, 41)	High consistency for EASE validity No direct cannabis studies 4/4 high-moderate quality
Phenomenological Overlap	Dissociation and self-disorders show experiential overlap but distinguishing features Schizophrenia: greater self-world confusion, automaticity	2 studies (33, 47)	Moderate consistency Theoretical + limited empirical High conceptual rigor
Cannabis & Self-Disturbance	THC and SPICE users show elevated self-disturbance markers *vs* non-users SPICE most severe Aberrant salience implicated	1 study (33)	Cannot assess consistency Single study High quality but preliminary
Endocannabinoid System	FEP shows reduced CB1 receptor availability in PFC/insula/PCC Disrupted endocannabinoid-receptor associations Elevated peripheral 2-AG	3 studies (38, 43, 44)	Consistent direction Small samples High-moderate quality
Neural Networks (DMN)	THC reduces DMN hyperconnectivity in patients Increases DMN-ECN anticorrelation Effects correlate with working memory	2 studies (7, 45)	Consistent mechanistic findings Small samples Moderate-high quality
Genetic Moderation	COMT Val carriers: increased risk with adolescent cannabis CNR1 polymorphisms interact with cannabis for psychosis onset	2 studies (36, 37)	High consistency Gene-environment interactions Both high quality
Functional Outcomes	Dissociation severity predicts functional decline (β=-0.34) Persistent cannabis + dissociation: worse hospitalization, adherence, social functioning	2 studies (32, 34)	High consistency Both longitudinal Both high quality
Treatment Response	Attenuated antipsychotic response in cannabis users Dissociation may show different trajectory than positive symptoms	1 study (46)	Cannot assess consistency Single moderate-quality study Preliminary

Consistency ratings based on direction and magnitude of effects across studies. High consistency, all studies show similar effects; Moderate, majority show similar effects with some variation; Low/unclear, conflicting findings or insufficient studies. DMN, default mode network; ECN, executive control network.

## 4 Discussion

This systematic review provides the first comprehensive synthesis of evidence examining relationships among cannabis use, dissociative experiences, and self-disturbance in first-episode psychosis. Several key findings emerge with theoretical and clinical implications: (1) cannabis use, particularly high-potency THC products, is consistently associated with elevated dissociative experiences in FEP patients, with clinically meaningful effect sizes and apparent dose-response relationships; (2) cannabis-related dissociation in FEP shares phenomenological features with both primary dissociative disorders and fundamental self-disturbances characteristic of schizophrenia spectrum pathology; (3) neurobiological evidence demonstrates endocannabinoid system dysregulation affecting brain networks critical for self-consciousness and reality monitoring; (4) dissociative phenomenology and self-disturbance in cannabis-using FEP patients predict poorer functional outcomes and symptom chronicity; and (5) significant methodological heterogeneity and gaps limit definitive causal conclusions while highlighting directions for future research.

### 4.1 Dissociation as a central feature of cannabis-associated psychosis

The consistent finding of elevated dissociative experiences among cannabis-using FEP patients represents an important phenomenological characterization of cannabis-associated psychosis. Effect sizes, with mean DES-II score differences of approximately 12 points between users and non-users, exceed standard deviation and proposed clinical significance thresholds (>30 on DES-II indicating pathological dissociation). This magnitude of association is comparable to or exceeds dissociation differences between other clinical populations and healthy controls, suggesting dissociative phenomenology constitutes a core rather than peripheral feature of cannabis-related psychosis.

The demonstration of dose-response relationships—with daily high-potency cannabis use associated with three-fold increased odds of significant dissociation compared to non-use—strengthens causal inference within observational study limitations. These findings align with experimental THC administration studies demonstrating dose-dependent dissociative effects (Mathew et al., 1993; 7), suggesting biological plausibility for observed associations.

However, several complexities warrant consideration. First, the persistence of dissociative symptoms in approximately one-quarter of patients despite cannabis cessation (32) suggests either enduring neurobiological alterations from cannabis exposure or, alternatively, that cannabis use may unmask pre-existing vulnerability to dissociative experiences rather than causing *de novo* dissociation. Distinguishing between these possibilities requires prospective studies assessing dissociation before cannabis initiation—a methodologically challenging but theoretically important research direction.

Second, the relationship between acute cannabis-induced dissociation and trait dissociative tendencies remains unclear. Does repeated experience of drug-induced depersonalization/derealization sensitize individuals to dissociative states, effectively ‘training’ dissociative responding? Or does baseline dissociative tendency predict greater subjective dissociative response to cannabis, creating a selection effect? Longitudinal studies with frequent assessments could disentangle state versus trait contributions.

Experimental THC studies in healthy volunteers support biological plausibility: Mathew et al. (15) demonstrated dose-dependent depersonalization, with temporal disintegration predicting severity (β=0.61, p<0.001). Freeman et al. (24) documented European cannabis potency increases during 2006-2016 (resin: 8.14%→17.22% THC; herbal: 5.00%→10.22%), providing context for understanding contemporary FEP patients’ THC exposure levels during the study period.

### 4.2 Cannabis, dissociation, and self-disturbance: phenomenological intersections

Perhaps the most theoretically significant finding is the apparent phenomenological convergence between cannabis-related dissociation and fundamental self-disturbances (ipseity disturbances) characteristic of schizophrenia spectrum disorders. Cannabis-using FEP patients demonstrate not only quantitatively greater dissociation but also qualitatively distinct phenomenology compared to primary dissociative disorders: greater self-world confusion, more severe first-person perspective disturbances, and experiential features overlapping with classical descriptions of self-disorders (Selbststörungen).

This convergence raises profound questions about the relationship between cannabis exposure and the core psychopathology of schizophrenia spectrum disorders. Classical phenomenological psychiatry (Jaspers, Schneider, Blankenburg) emphasized disturbances of self-experience as the fundamental disorder from which other schizophrenia symptoms emerge. Contemporary phenomenological research has operationalized this framework through instruments like the EASE, demonstrating that self-disorders aggregate specifically in schizophrenia spectrum conditions, predict psychosis onset, and show genetic loading (13).

The ipseity-disturbance model (10) proposes two complementary aspects of self-disorder: hyper reflexivity (exaggerated focal awareness of processes normally experienced tacitly) and diminished self-affection (weakened sense of existing as subject of awareness). Cannabis intoxication phenomenology strikingly parallels these dimensions. Acute cannabis effects frequently include heightened awareness of normally automatic mental processes (hyperreflexivity-like phenomena), simultaneous sense of detachment or depersonalization (diminished self-affection-like phenomena), and altered ‘grip’ on reality with disturbances of salience and affordance (disturbed hold-like phenomena).

This phenomenological similarity suggests three non-mutually-exclusive possibilities. First, repeated cannabis exposure might causally contribute to developing ipseity disturbance in vulnerable individuals, essentially exogenously inducing core schizophrenia spectrum psychopathology. Second, individuals with subclinical or prodromal ipseity disturbance might be particularly sensitive to cannabis’s phenomenological effects, experiencing more pronounced dissociative responses. Third, cannabis and endogenous vulnerability factors might interact synergistically, with cannabis exposure accelerating or amplifying emerging self-disturbance in genetically or developmentally vulnerable individuals.

The third possibility appears most consistent with available evidence. Genetic studies demonstrating gene-environment interactions (COMT, CNR1 polymorphisms moderating cannabis-psychosis associations) support biological vulnerability × environmental exposure models. Epidemiological evidence that only a minority of cannabis users develop persistent psychosis despite widespread exposure suggests specific vulnerability factors. The finding that childhood trauma amplifies cannabis-related dissociation and self-disturbance (41) further supports diathesis-stress frameworks wherein cannabis acts as environmental precipitant in vulnerable individuals.

The ipseity-disturbance model (10) proposes schizophrenia involves hyper reflexivity (exaggerated self-consciousness) and diminished self-affection (weakened sense of existing as subject). Cannabis intoxication phenomenology parallels both dimensions: heightened awareness of normally tacit processes (hyperreflexivity-like) and simultaneous detachment (diminished self-affection-like). The environmental sensitivity of EASE measures demonstrated by Haug et al. (41)—correlations with childhood trauma (r=0.48, p<0.001)—suggests self-disturbance can respond to environmental factors, supporting biological plausibility for cannabis effects.

### 4.3 Neurobiological underpinnings: endocannabinoid system and self-consciousness networks

Neurobiological findings provide mechanistic insights into phenomenological observations. The demonstration of altered CB1 receptor availability and disrupted endocannabinoid-receptor associations specifically in FEP patients (38) suggests fundamental endocannabinoid system dysregulation beyond acute drug effects. Particularly significant is the localization of these alterations to posterior cingulate cortex—a critical node in the default mode network (DMN) that subserves self-referential processing and mind-wandering, processes intimately related to phenomenological self-experience. Beyond the endocannabinoid system, dopaminergic mechanisms also contribute to cannabis-related phenomenological disturbances. Bloomfield et al. (17) demonstrated that aberrant salience attribution—a dopamine-mediated process central to psychosis—correlates significantly with cannabis-induced psychotic symptom severity (r=0.61, p=0.04). Critically, cannabis users showed disrupted relationships between striatal dopamine synthesis capacity and salience processing compared to controls, suggesting that chronic cannabis exposure fundamentally alters reward-prediction and salience mechanisms that may underlie both psychotic symptoms and self-disturbance. The neurobiological substrate for these salience alterations appears to involve both reduced striatal dopamine synthesis capacity (39) and disrupted endocannabinoid-dopamine interactions. Chronic cannabis users show dose-dependent reductions in striatal dopamine synthesis, with earlier onset of use predicting greater dopaminergic dysfunction—a pattern consistent with neurodevelopmental vulnerability models of cannabis-psychosis associations. The dissociation between reduced baseline dopamine synthesis and aberrant salience processing in cannabis users suggests complex, non-linear dopaminergic mechanisms where chronic receptor downregulation may paradoxically enhance sensitivity to phasic dopamine signaling during acute intoxication or stress.

The DMN has emerged as perhaps the primary neural correlate of self-consciousness and minimal self-experience. DMN activity characterizes rest and internally-directed cognition, with core hubs in medial prefrontal cortex and posterior cingulate/precuneus. Critically, these regions show among the highest CB1 receptor densities in the brain. THC administration acutely disrupts DMN connectivity, with connectivity disruption correlating with subjective dissociative experiences. This provides direct mechanistic link between cannabinoid receptor activation and phenomenological self-disturbance.

Furthermore, the finding that FEP patients show baseline CB1 receptor reductions in DMN regions suggests a ‘double hit’ scenario. Endogenous endocannabinoid system dysfunction may already compromise self-related processing in psychosis-vulnerable individuals. Exogenous cannabinoid exposure then further perturbs this already-compromised system, producing particularly pronounced phenomenological disturbances. This model could explain both why cannabis users in general rarely develop persistent dissociation/self-disturbance (intact baseline systems tolerate perturbation) and why FEP patients show such vulnerability (compromised baseline makes system unstable to additional challenge). The insula represents another neurobiologically and phenomenologically critical structure. Highly CB1-receptor-dense, the insula integrates interoceptive signals into conscious awareness and contributes fundamentally to embodied self-experience. Anterior insula particularly supports sense of body ownership and agency. Alterations in insular function have been documented in both dissociative disorders and schizophrenia spectrum conditions. Cannabis effects on insular processing remain understudied but represent important mechanistic pathway potentially linking cannabinoid exposure to disturbed embodiment—a core feature of both dissociation and self-disorders. The temporal dimension of cannabis effects adds additional complexity. Acute THC produces transient dissociation and DMN disruption, typically resolving within hours. However, chronic heavy cannabis use may produce enduring alterations. Preclinical studies demonstrate CB1 receptor downregulation and desensitization following chronic cannabinoid exposure. Whether such alterations occur in human cortical regions relevant to self-consciousness, and whether they normalize with prolonged abstinence, remains incompletely characterized. The persistence of dissociative symptoms in some individuals months after cannabis cessation suggests potential long-lasting neurobiological changes, though longitudinal neuroimaging studies directly addressing this question are lacking. These findings suggest mechanistic pathways linking cannabis to dissociation and self-disturbance. CB1 receptor alterations in FEP (38) localize to posterior cingulate cortex—a DMN node critical for self-referential processing. Whitfield-Gabrieli et al. (45) demonstrated THC acutely disrupts DMN connectivity in schizophrenia patients, linking cannabinoid effects to neural networks supporting self-consciousness. This suggests a ‘double hit’: endogenous endocannabinoid dysfunction in FEP plus exogenous cannabis exposure may synergistically compromise self-related processing. Aberrant salience may mediate cannabis effects on both dissociation and self-disturbance. Cannabis-associated salience alterations (17, 33) produce experiences of altered significance overlapping with depersonalization (altered self-significance) and self-disorders (disturbed sense of salience for the experiencing subject), representing a potential mechanistic pathway.

### 4.4 Clinical implications and assessment recommendations

The demonstrated associations between cannabis use, dissociation, and self-disturbance carry significant clinical implications for FEP assessment and intervention. Current standard psychiatric assessment in early psychosis typically emphasizes positive symptoms (hallucinations, delusions), negative symptoms, and functional capacity, with less systematic attention to dissociative phenomenology or subtle self-disturbances. Our findings suggest comprehensive assessment should incorporate specific evaluation of dissociative experiences and anomalous self-experience, particularly in cannabis-using patients.

From a prognostic perspective, clinicians should recognize that pronounced dissociative phenomenology and self-disturbance may indicate greater illness severity and poorer functional trajectory, independent of positive symptom burden. This suggests potential value in treatment intensification for patients presenting with this phenomenological profile. However, optimal intervention strategies for cannabis-related dissociation remain unclear from available literature.

Standard antipsychotic treatment appears to address positive symptoms but may insufficiently target dissociative phenomenology. This suggests potential role for adjunctive interventions specifically addressing dissociation and self-disturbance. Psychotherapeutic approaches with evidence in dissociative disorders (trauma-focused therapy when appropriate, mindfulness-based interventions, sensorimotor psychotherapy targeting embodiment) warrant investigation in cannabis-using FEP populations. Given theoretical links between self-disorders and metacognitive deficits, metacognitive training approaches deserve exploration.

Regarding substance use treatment, our findings underscore importance of cannabis-specific interventions integrated into FEP care. Evidence suggests cannabis cessation associates with dissociation reduction in most patients, highlighting cessation as therapeutic target. However, approximately 25% maintain dissociation despite abstinence, suggesting these patients require additional dissociation-focused interventions. Motivational enhancement therapy addressing both cannabis use and dissociative distress may prove valuable, though this requires empirical investigation.

### 4.5 Methodological considerations and limitations

Several methodological limitations affect interpretation and confidence in conclusions. First, the predominance of cross-sectional studies (55% of included studies) limits causal inference regarding directional relationships between cannabis, dissociation, and self-disturbance. While experimental THC studies demonstrate acute causation of dissociative experiences, these controlled administrations may not generalize to naturalistic chronic use patterns. Longitudinal observational studies provide stronger causal inference but remain vulnerable to confounding.

Second, reliance on self-reported cannabis use without biochemical verification in 64% of studies introduces potential information bias. Cannabis users may underreport use due to stigma or legal concerns, while psychotic patients may have impaired recall or insight. However, the consistency of findings across studies using different assessment methods (self-report, structured interview, urinalysis) provides some reassurance about robustness of associations.

Third, heterogeneity in dissociation and self-disturbance assessment complicates synthesis. While standardized instruments like DES-II enable some quantitative comparison, diverse assessment approaches (PANSS dissociation items, clinical assessment, EASE) measure potentially different constructs. Dissociation itself encompasses multiple dimensions (depersonalization, derealization, absorption, amnesia), with subscale analyses revealing that cannabis particularly associates with depersonalization/derealization rather than other dissociative dimensions. Future studies should employ comprehensive multidimensional dissociation assessment.

Fourth, limited control for potential confounding variables, particularly polysubstance use, affects many studies. Cannabis users in FEP populations frequently use tobacco (co-occurrence 50-90%), alcohol, and stimulants, each of which may independently contribute to dissociative experiences. Tobacco affects cholinergic and dopaminergic systems relevant to dissociation; stimulants can directly induce dissociative and psychotic symptoms; alcohol produces acute dissociative effects. Only a minority of included studies systematically assessed and controlled for concurrent tobacco, alcohol, and stimulant use, with many reporting “cannabis effects” without adequately differentiating cannabis-specific contributions from polysubstance patterns. This limitation is particularly significant given that polysubstance use is normative in cannabis-using FEP populations. Additionally, childhood trauma, family history of psychosis, and premorbid personality traits may confound relationships, with few studies comprehensively controlling for multiple confounders simultaneously. Inadequate trauma assessment is particularly problematic given established trauma-dissociation and trauma-psychosis associations.

Fifth, establishing causality between cannabis use and dissociative experiences faces several fundamental challenges. While our review demonstrates robust associations and dose-response relationships, multiple alternative explanations warrant consideration. Reverse causality represents an important possibility: individuals experiencing early dissociative symptoms or anomalous self-experiences may self-medicate with cannabis, producing associations even without causal cannabis effects. Genetic confounding is another challenge, as shared genetic liability may predispose individuals to both cannabis use and dissociative/psychotic experiences independently. Common environmental factors—particularly childhood trauma, urban upbringing, and social adversity—increase risk for both cannabis use and dissociative psychopathology, potentially generating spurious associations. Bidirectional relationships further complicate inference: cannabis may increase dissociation, which influences continued use patterns, creating feedback loops difficult to disentangle observationally. While dose-response gradients and experimental THC studies support causal hypotheses, definitive causal conclusions require stronger designs (e.g., Mendelian randomization, discordant twin studies, intensive longitudinal analyses with time-lagged effects) currently absent from this literature.

Sixth, the scarcity of studies specifically examining self-disturbance (EASE-assessed anomalous self-experience) in relation to cannabis use represents a significant gap. While phenomenological convergence between dissociation and self-disorders provides theoretical rationale for linking cannabis to ipseity disturbance, direct empirical evidence remains limited. Studies combining EASE assessment with detailed cannabis use characterization in FEP populations would substantially advance understanding.

Seventh, neurobiological studies, while providing mechanistic insights, remain limited in number and scope. Dickens et al. (38) provides important PET neuroimaging evidence, but small sample size (n=28 FEP patients across two cohorts) limits generalizability. Furthermore, the study did not assess dissociation or self-disturbance phenomenology, preventing direct linking of neurobiological findings to experiential alterations. Future research integrating neuroimaging, peripheral biomarkers, and comprehensive phenomenological assessment would optimally elucidate mechanisms.

We did not perform quantitative meta-analysis due to substantial heterogeneity in study designs, cannabis exposure definitions, and dissociation assessment methods. While this limits precise effect size estimation, narrative synthesis allowed for comprehensive integration of diverse evidence types including neuroimaging and phenomenological studies that would have been excluded from meta-analysis.

### 4.6 Future research directions

Several high-priority research directions emerge:

Longitudinal phenotyping studies should follow individuals at clinical high risk for psychosis, assessing dissociation and self-disturbance before psychosis onset, during transition, and throughout early course. Notably, existing CHR studies examining cannabis use and psychosis transition have yielded inconsistent findings, with some demonstrating increased conversion rates among cannabis users while others find no significant associations. Integrating comprehensive phenomenological assessment (dissociation, self-disturbance) with detailed cannabis use characterization may help explain these heterogeneous findings by identifying specific experiential markers that moderate cannabis-psychosis relationships and clarify whether dissociative phenomenology represents a prodromal feature, mediating mechanism, or independent dimension.

Mechanistic neuroimaging studies combining functional connectivity analysis (particularly DMN assessment), CB1 receptor neuroimaging, peripheral endocannabinoid measurement, and comprehensive phenomenological evaluation would optimally link neural mechanisms to experiential alterations. Studies should examine both acute cannabis effects and enduring alterations in chronic users, with longitudinal assessment following cannabis cessation. Genetic studies should examine whether polymorphisms in genes related to endocannabinoid signaling (CNR1, FAAH, MGLL), dopaminergic function (COMT, DRD2), and stress response (FKBP5, NR3C1) moderate cannabis effects on dissociation and self-disturbance. Gene × environment × phenotype models could identify specific vulnerability profiles.

Intervention trials should examine whether cannabis-specific substance use interventions reduce dissociative symptoms and improve functional outcomes in FEP. Additionally, trials of dissociation-focused psychotherapies (trauma therapy when indicated, mindfulness-based interventions, sensorimotor approaches) warrant investigation. Given preliminary CBD evidence in psychosis generally, studies examining whether CBD co-administration mitigates THC-related dissociation deserve exploration.

Phenomenological research employing rigorous qualitative methods should deeply characterize experiential qualities distinguishing cannabis-related from primary dissociation and self-disorders. Micro-phenomenological interviewing capturing fine-grained experiential structure could reveal subtle distinctions currently obscured by standardized instruments. Such research would refine phenomenological taxonomy and potentially identify novel therapeutic targets. Developmental studies should examine whether adolescent-onset cannabis use specifically influences self-disturbance development, given adolescence as critical period for both self-identity formation and vulnerability to psychosis onset. Comparing adolescent-onset versus adult-onset cannabis users on dissociation and self-disturbance measures could reveal developmentally-sensitive periods.

### 4.7 Rationale for including studies without direct cannabis-self-disturbance assessment

A methodological consideration requiring explicit justification concerns our inclusion of studies that examined self-disturbance in FEP populations without specifically analyzing cannabis use effects (e.g., 14, 35, 41, 42, 47). This inclusion strategy was deliberate and theoretically motivated for several reasons.

Establishing the validity and clinical significance of self-disturbance bmeasures in FEP populations constitutes an essential foundation for subsequently examining whether cannabis modulates these phenomena. The EASE instrument psychometric properties (35), predictive validity for psychosis conversion (14), and associations with functional outcomes (42) provide critical context for interpreting cannabis-related self-disturbance findings. Without understanding baseline self-disturbance phenomenology in FEP generally, cannabis-specific effects cannot be meaningfully characterized.

Phenomenological overlap between dissociative experiences and self-disorders required theoretical clarification independent of cannabis effects. Sass et al.’s (47) comparative analysis of depersonalization disorder and schizophrenia-spectrum self-disorders established phenomenological distinctions essential for interpreting whether cannabis-related dissociation represents primary dissociative phenomena or manifestations of fundamental ipseity disturbance. This theoretical framework informed our synthesis of cannabis-specific findings.

Studies documenting environmental sensitivity of self-disturbance measures particularly trauma associations (41)—provide crucial evidence that self-disorders respond to environmental factors, supporting biological plausibility for cannabis effects. If self-disturbance were purely endogenous and immutable, environmental exposures like cannabis would be less likely to modulate these experiences. Demonstrating environmental sensitivity in FEP populations (even for non-cannabis factors) strengthens the conceptual foundation for hypothesizing cannabis-self-disturbance interactions.

Neurobiological studies examining endocannabinoid system alterations in FEP patients** (38, 43, 44) identified CB1 receptor dysregulation and peripheral endocannabinoid changes in FEP populations independent of cannabis exposure history. These findings demonstrate that the endocannabinoid system—the primary target of cannabis effects—is already disrupted in early psychosis. This provides mechanistic rationale for why exogenous cannabinoid exposure might particularly impact self-experience in FEP populations: cannabis perturbs an already-dysregulated system in regions critical for self-consciousness (posterior cingulate cortex, prefrontal cortex, insula).

Finally, from a methodological transparency perspective, including validation studies and neurobiological investigations enriches our understanding while acknowledging that direct evidence specifically linking cannabis use to self-disturbance assessed via EASE remains limited. This limitation is explicitly noted in our quality assessment and GRADE ratings, where self-disturbance outcomes received “low” certainty ratings precisely due to limited direct evidence. Our approach prioritizes comprehensive theoretical contextualization over narrow empiricism, recognizing that understanding complex phenomenological-neurobiological relationships requires integrating diverse evidence streams.

In summary, the inclusion of FEP studies without cannabis-specific analyses was not a methodological compromise but rather a deliberate strategy to: (1) establish psychometric foundations; (2) clarify phenomenological taxonomy; (3) demonstrate environmental sensitivity of self-disturbance; (4) elucidate endocannabinoid system dysregulation providing mechanistic plausibility; and (5) transparently acknowledge evidence limitations. Future research directly examining cannabis effects on EASE-assessed self-disturbance represents a critical priority emerging from this synthesis.

## 5 Conclusion

This systematic review demonstrates robust associations between cannabis use and dissociative experiences in first-episode psychosis (effect sizes: d=0.97-1.12), with emerging evidence of phenomenological overlap with fundamental self-disturbances characteristic of schizophrenia spectrum disorders. Daily high-potency cannabis use confers three-fold increased odds of clinically significant dissociation compared to non-use, with dose-response patterns strengthening causal inference. Neurobiological evidence reveals endocannabinoid system dysregulation in FEP patients, particularly reduced CB1 receptor availability in regions critical for self-consciousness (posterior cingulate cortex, prefrontal cortex, insula). This provides mechanistic plausibility for cannabis’s pronounced dissociative effects in psychosis-vulnerable individuals: exogenous cannabinoids perturb an already-compromised system. Clinically, comprehensive phenomenological assessment incorporating dissociative symptomatology (DES-II, CADSS) and anomalous self-experience (EASE when feasible) should become standard in cannabis-using FEP patients. Cannabis cessation represents a critical therapeutic target, with approximately 75% showing dissociation reduction following abstinence, though the 25% with persistent symptoms require adjunctive dissociation-focused interventions. Critical research priorities include: (1) prospective studies assessing self-disturbance before and after cannabis initiation in clinical high-risk populations; (2) neuroimaging integrating CB1 receptor imaging, functional connectivity, and phenomenological assessment; (3) intervention trials examining dissociation-focused psychotherapies; and (4) genetic studies of polymorphisms moderating cannabis effects on self-disturbance. As cannabis potency increases globally and legalization expands availability, understanding these phenomenological dimensions becomes urgent. This synthesis demonstrates that cannabis-psychosis relationships extend beyond diagnostic risk to encompass specific experiential alterations—dissociation and self-disturbance— with independent prognostic and therapeutic significance, requiring integrated phenomenological and neurobiological approaches in future research and clinical practice.
