# Supplementary material for: Self-disturbance in first-episode psychosis: Theoretical framework and potential cannabis interactions - a systematic review
Source: Front Psychiatry. 2026 Jan 5;16:1733254. doi: 10.3389/fpsyt.2025.1733254 (PMC12812679; doi:10.3389/fpsyt.2025.1733254)
Supplement: Supplementary file 1 [file Table1.docx]

# **Appendix 1. Database Search Results and Study Selection**

| **Database** | **Search Date** | **Initial Results** | **After Deduplication** | **After Screening** |
| --- | --- | --- | --- | --- |
| **PubMed/MEDLINE** | March 15, 2024 | 342 | 287 | 38 |
| **Scopus** | March 15, 2024 | 487 | 301 | 52 |
| **PsycINFO** | March 16, 2024 | 231 | 178 | 24 |
| **Web of Science** | March 16, 2024 | 187 | 95 | 13 |
| **Grey Literature** |  |  |  |  |
| ClinicalTrials.gov | March 17, 2024 | 8 | - | 0 |
| WHO ICTRP | March 17, 2024 | 3 | - | 0 |
| Manual/Citation Screening | March 18-20, 2024 | 10 | - | 10 |
| **TOTAL** | **-** | **1,268** | **861** | **137** |
| **Full-text assessed** | **-** | **-** | **-** | **127** |
| **Studies included in review** | **-** | **-** | **-** | **23** |

# **Appendix 2. Reasons for Exclusion at Full-Text Review (n=104)**

| **Exclusion Reason** | **n** | **%** | **Representative Examples** |
| --- | --- | --- | --- |
| No specific assessment of dissociation or self-disturbance | 38 | 36.5 | PANSS total only; general symptom scales without phenomenology |
| Chronic psychosis (>5 years) without separate FEP analysis | 21 | 27.9 | Mixed samples; no subgroup data for recent-onset |
| Cannabis not differentiated from other substance use | 14 | 13.5 | Polysubstance use as single category; no cannabis-specific data |
| Wrong study type (review, case report n<10, abstract only) | 10 | 9.6 | Systematic reviews; conference abstracts; editorials |
| Synthetic cannabinoids only (no natural cannabis data) | 6 | 5.8 | SPICE/K2 studies without THC comparison |
| Insufficient data for extraction (authors non-responsive) | 4 | 3.8 | Missing statistics; incomplete reporting |
| Non-English without available translation | 3 | 2.9 | 2 German, 1 French |
| **TOTAL** | **96** | **92.3** | - |

*Note: Total excludes 23 studies that were included in the final review (127 full-text assessed - 23 included = 104 excluded).*

# **Appendix 3. Assessment Instruments Used Across Included Studies (N=22)**

| **Instrument Type** | **Instrument Name** | **Studies (n)** | **Key Features / Scoring** |
| --- | --- | --- | --- |
| **Dissociation Measures** |  |  |  |
|  | DES-II | 6 | 28 items, 0-100 scale; ≥30 pathological threshold; subscales: amnesia, absorption, depersonalization/derealization |
|  | Cambridge Depersonalization Scale | 2 | 29 items, frequency and duration ratings; focuses on depersonalization/derealization specifically |
|  | PANSS Dissociation Items | 3 | Selected items: G12 (lack of judgment), G9 (unusual thought content); 1-7 Likert scale |
|  | CADSS | 2 | Clinician-Administered Dissociative States Scale; 27 items, 0-4 scale; acute dissociative states |
|  | Psychotomimetic States Inventory (PSI) | 1 | Cannabis-induced psychotic symptoms; validated scale for acute effects; multiple dimensions including perceptual alterations and cognitive disorganization |
| **Self-Disturbance Measures** |  |  |  |
|  | EASE | 5 | Examination of Anomalous Self-Experience; 57 items in 5 domains; 0-4 severity; semi-structured interview; excellent reliability (kappa>0.80) |
|  | Salience Attribution Test (SAT) | 2 | Probabilistic reward-learning task measuring aberrant salience; implicit and explicit measures; adaptive motivational salience vs maladaptive aberrant salience |
|  | BSABS | 1 | Bonn Scale for Assessment of Basic Symptoms; self-disturbance items subset |
| **Cannabis Assessment** |  |  |  |
|  | SCID-I CUD Module | 4 | Structured Clinical Interview; DSM-IV Dependence or DSM-5 Cannabis Use Disorder diagnosis |
|  | Structured Interview | 10 | Frequency (never/occasional/weekly/daily), duration, age onset, potency when available, dependency/abuse status |
|  | Urinalysis | 3 | THC metabolite detection; confirms recent use (2-4 weeks for regular users) |
|  | Self-Report Questionnaire | 6 | Lifetime use, frequency patterns, self-reported potency |
| **Psychotic Symptoms** |  |  |  |
|  | PANSS | 12 | Positive and Negative Syndrome Scale; 30 items, 1-7 scale; positive, negative, general subscales |
|  | BPRS | 3 | Brief Psychiatric Rating Scale; 18-24 items depending on version |
| **Functional Outcomes** |  |  |  |
|  | GAF | 5 | Global Assessment of Functioning; 0-100 scale; symptom severity and social/occupational functioning |
|  | SOFAS | 3 | Social and Occupational Functioning Assessment Scale; 0-100; functioning independent of symptoms |
| **Neurobiological Measures** |  |  |  |
|  | CB1 Receptor PET | 2 | Positron emission tomography with CB1 radioligands; measures receptor availability; regions: PFC, insula, PCC |
|  | [18F]-DOPA PET | 2 | Dopamine synthesis capacity measurement; Kicer (influx rate constant); striatal subregions (associative, limbic, sensorimotor) |
|  | Peripheral Endocannabinoids | 1 | Plasma 2-AG and anandamide levels; ELISA or mass spectrometry |
|  | fMRI Resting-State | 2 | Default mode network connectivity; DMN-ECN anticorrelation; regional activation patterns |
| **Genetic Markers** |  |  |  |
|  | COMT Val158Met | 1 | Catechol-O-methyltransferase polymorphism; dopamine catabolism; gene-environment interaction |
|  | CNR1 (rs2023239) | 1 | Cannabinoid receptor 1 gene polymorphism; CB1 receptor expression and function |

*Note: Studies may have used multiple instruments. SAT = Salience Attribution Test; PSI = Psychotomimetic States Inventory; [18F]-DOPA = 3,4-dihydroxy-6-[18F]fluoro-L-phenylalanine; Kicer = influx rate constant; PET = positron emission tomography; fMRI = functional magnetic re*

**Appendix 4. Individual Study Quality Assessment Scores**

| **Study** | **Selection (0-4)** | **Comparability (0-2)** | **Outcome (0-3)** | **Total (0-9)** | **Quality Rating** |
| --- | --- | --- | --- | --- | --- |
| Ricci et al., 2021 | 4 | 2 | 2 | 8 | High |
| Quattrone et al., 2020 | 3 | 2 | 3 | 8 | High |
| Di Forti et al., 2019 | 4 | 2 | 2 | 8 | High |
| Freeman et al., 2019 | 2 | 1 | 3 | 6 | Moderate |
| Núñez & Gurpegui, 2002 | 2 | 1 | 3 | 6 | Moderate |
| Mathew et al., 1999 | - | - | - | RoB: Low | High (RCT) |
| Møller et al., 2011 | 3 | 2 | 2 | 7 | High |
| Nelson et al., 2013 | 4 | 2 | 2 | 8 | High |
| Haug et al., 2012 | 3 | 1 | 2 | 6 | Moderate |
| Haug et al., 2014 | 3 | 2 | 2 | 7 | High |
| Sass et al., 2013 | - | - | - | N/A | Theoretical |
| Sass & Parnas, 2003 | - | - | - | N/A | Theoretical |
| Ricci et al., 2024 | 4 | 2 | 2 | 8 | High |
| Dickens et al., 2020 | 3 | 2 | 2 | 7 | High |
| Bioque et al., 2013 | 3 | 1 | 2 | 6 | Moderate |
| Ceccarini et al., 2013 | 3 | 2 | 2 | 7 | High |
| Whitfield-Gabrieli et al., 2017 | 2 | 1 | 2 | 5 | Moderate |
| Bloomsfield, 2016 | 3 | 3 | 2 | 8 | High |
| Bloomsfield, 2014 | 3 | 1 | 3 | 7 | High |
| D'Souza et al., 2004 | - | - | - | RoB: Low | High (RCT) |
| Caspi et al., 2005 | 4 | 2 | 2 | 8 | High |
| Colizzi et al., 2015 | 3 | 2 | 2 | 7 | High |
| Nelson et al., 2012 | 4 | 2 | 2 | 8 | High |
| Patel et al., 2016 | 2 | 2 | 2 | 6 | Moderate |
|  |  |  |  |  |  |
| **Quality Distribution** |  |  | **High: 11 (55%)** | **Moderate: 7 (35%)** | **Theoretical: 2 (10%)** |

*Note: NOS = Newcastle-Ottawa Scale for observational studies (0-9 points). RoB = Cochrane Risk of Bias tool for experimental studies. High quality = 7-9 points or Low RoB; Moderate = 4-6 points or Some concerns; Theoretical studies not scored.*

**Appendix 5. Effect Sizes for Primary Outcomes by Study**

| **Study** | **Outcome** | **Effect Size** | **95% CI** | **p-value** |
| --- | --- | --- | --- | --- |
| **Ricci et al., 2021** |  |  |  |  |
|  | DES-II baseline difference | d = 1.02 | 0.54 to 1.50 | <0.01 |
|  | DES-II 8-month difference | d = 0.78 | 0.32 to 1.24 | <0.01 |
|  | GAF decline (cannabis users) | β = -0.34 | -0.56 to -0.12 | 0.006 |
| **Quattrone et al., 2020** |  |  |  |  |
|  | Continued use → dissociation | β = 0.42 | 0.24 to 0.60 | <0.001 |
|  | Cessation → DES-II reduction | MD = -8.3 | -11.5 to -5.1 | <0.001 |
|  | Hospitalization risk | RR = 2.34 | 1.47 to 3.72 | <0.001 |
| **Di Forti et al., 2019** |  |  |  |  |
|  | Daily high-potency → dissociation | OR = 3.21 | 2.14 to 4.82 | <0.001 |
|  | High vs low potency (PANSS) | d = 1.15 | 0.89 to 1.41 | <0.001 |
| **Núñez & Gurpegui, 2002** |  |  |  |  |
|  | Depersonalization prevalence | OR = 4.50 | 1.58 to 12.81 | 0.004 |
|  | Derealization prevalence | OR = 6.43 | 2.11 to 19.58 | 0.001 |
| **Mathew et al., 1999** |  |  |  |  |
|  | THC → depersonalization | d = 1.28 | 0.78 to 1.78 | <0.001 |
|  | Temporal perception mediation | β = 0.61 | 0.38 to 0.84 | <0.001 |
| **Nelson et al., 2013** |  |  |  |  |
|  | EASE → conversion | OR = 3.8/SD | 1.9 to 7.6 | <0.001 |
| **Haug et al., 2012** |  |  |  |  |
|  | EASE ↔ childhood trauma | r = 0.48 | 0.21 to 0.69 | <0.001 |
| **Dickens et al., 2020** |  |  |  |  |
|  | CB1 availability ↔ symptoms | r = -0.54 | -0.76 to -0.23 | 0.003 |
| **D'Souza et al., 2004** |  |  |  |  |
|  | Temporal → depersonalization | Sobel z = 2.34 | - | 0.019 |
| **Caspi et al., 2005** |  |  |  |  |
|  | COMT Val × cannabis | OR = 10.9 | 2.2 to 54.1 | <0.01 |

*Abbreviations: d = Cohen's d (standardized mean difference); β = standardized regression coefficient; MD = mean difference; OR = odds ratio; RR = relative risk; r = correlation coefficient. DES-II = Dissociative Experiences Scale-II; GAF = Global Assessment of Functioning; PANSS = Positive and Negative Syndrome Scale; EASE = Examination of Anomalous Self-Experience; CB1 = cannabinoid receptor type 1; COMT = catechol-O-methyltransferase.*

**Appendix 6. List of Abbreviations**

| **Abbreviation** | **Full Term** |
| --- | --- |
| 2-AG | 2-Arachidonoylglycerol (endocannabinoid) |
| ASE | Anomalous Self-Experience |
| BPRS | Brief Psychiatric Rating Scale |
| BSABS | Bonn Scale for Assessment of Basic Symptoms |
| CADSS | Clinician-Administered Dissociative States Scale |
| CB1 | Cannabinoid Receptor Type 1 |
| CDS | Cambridge Depersonalization Scale |
| CHR | Clinical High-Risk (for psychosis) |
| CI | Confidence Interval |
| CNR1 | Cannabinoid Receptor 1 gene |
| COMT | Catechol-O-Methyltransferase gene |
| CUD | Cannabis Use Disorder |
| DES / DES-II | Dissociative Experiences Scale / Second Edition |
| DMN | Default Mode Network |
| DSM | Diagnostic and Statistical Manual of Mental Disorders |
| EASE | Examination of Anomalous Self-Experience |
| ECN | Executive Control Network |
| FEP | First-Episode Psychosis |
| fMRI | Functional Magnetic Resonance Imaging |
| GAF | Global Assessment of Functioning |
| GRADE | Grading of Recommendations Assessment, Development and Evaluation |
| ICD | International Classification of Diseases |
| ICTRP | International Clinical Trials Registry Platform (WHO) |
| NOS | Newcastle-Ottawa Scale (quality assessment) |
| OR | Odds Ratio |
| PANSS | Positive and Negative Syndrome Scale |
| PCC | Posterior Cingulate Cortex |
| PET | Positron Emission Tomography |
| PFC | Prefrontal Cortex |
| PRISMA | Preferred Reporting Items for Systematic Reviews and Meta-Analyses |
| PROSPERO | International Prospective Register of Systematic Reviews |
| RCT | Randomized Controlled Trial |
| RoB | Risk of Bias (Cochrane tool) |
| RR | Relative Risk |
| SCID | Structured Clinical Interview for DSM |
| SD | Standard Deviation |
| SMD | Standardized Mean Difference |
| SOFAS | Social and Occupational Functioning Assessment Scale |
| SPICE | Synthetic Cannabinoids (street name) |
| THC | Delta-9-Tetrahydrocannabinol (main psychoactive cannabinoid) |
| UHR | Ultra High-Risk (for psychosis) |
